# Supplementary material for: powerTCR: A model-based approach to comparative analysis of the clone size distribution of the T cell receptor repertoire
Source: PLoS Comput Biol. 2018 Nov 28;14(11):e1006571. doi: 10.1371/journal.pcbi.1006571 (PMC6287877; doi:10.1371/journal.pcbi.1006571)
Supplement: S1 Text — For each real data sample, a plot analagous to Fig 1A is provided. (PDF) [file pcbi.1006571.s001.pdf]

Supplementary file 1 — Visualization of all model fits

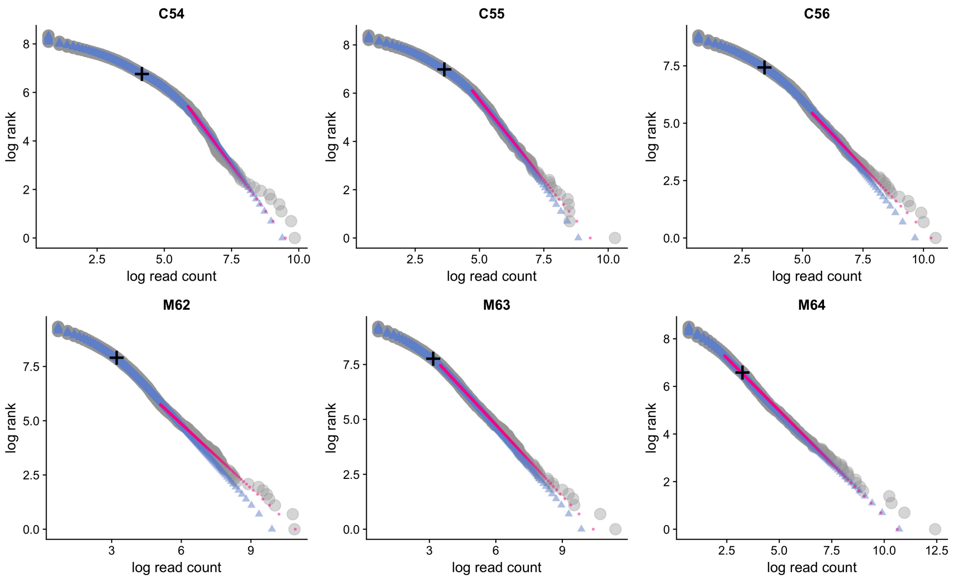

Figure A: Plots of all empirical murine breast cancer data and the fitted curves based on the Desponds method (pink) and our method (blue). The cross marks the threshold estimated by our method.

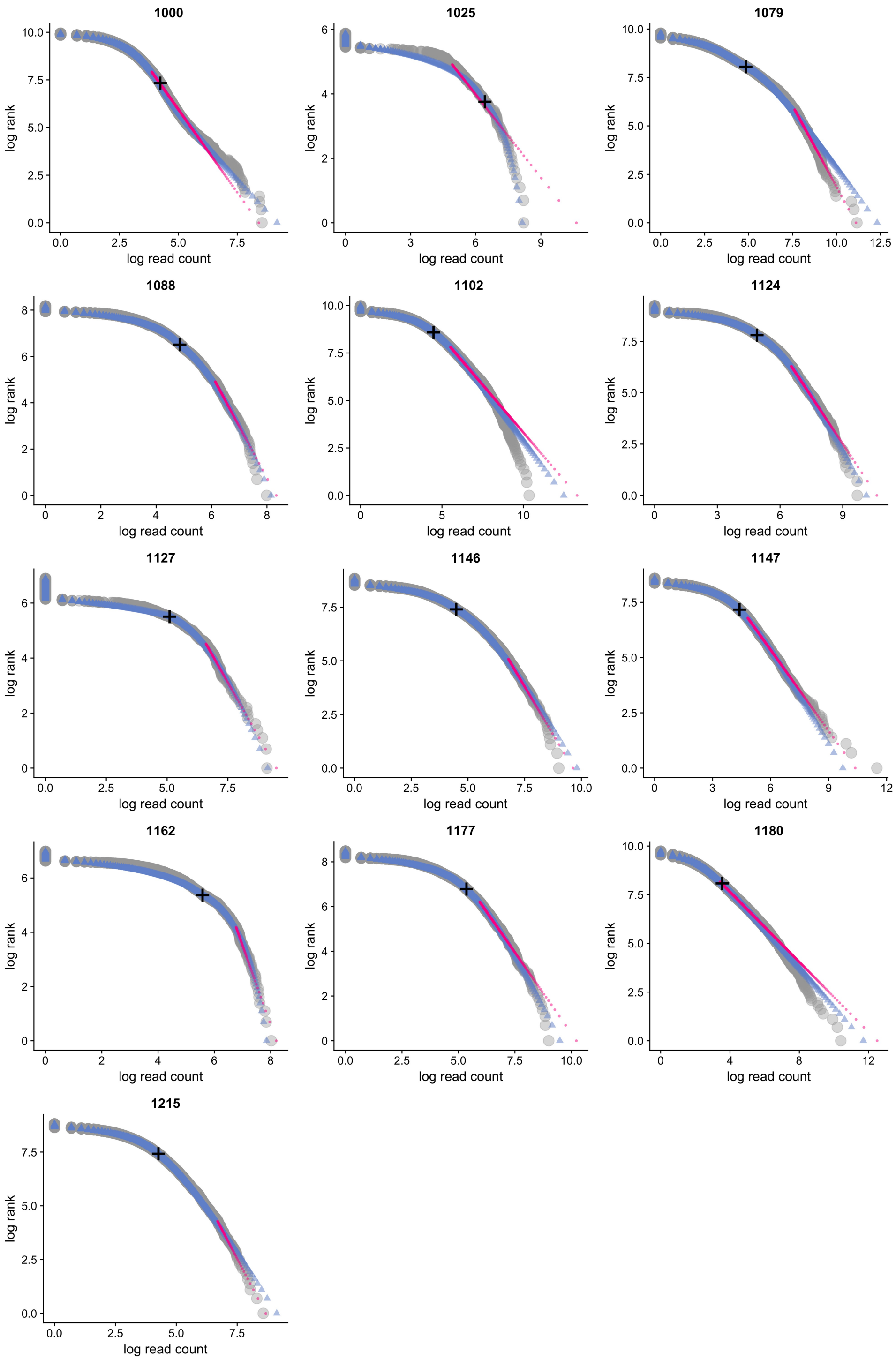

Figure B: Plots of all empirical Sarcoidosis patient data and the fitted curves based on the Desponds method (pink) and our method (blue). The cross marks the threshold estimated by our method.

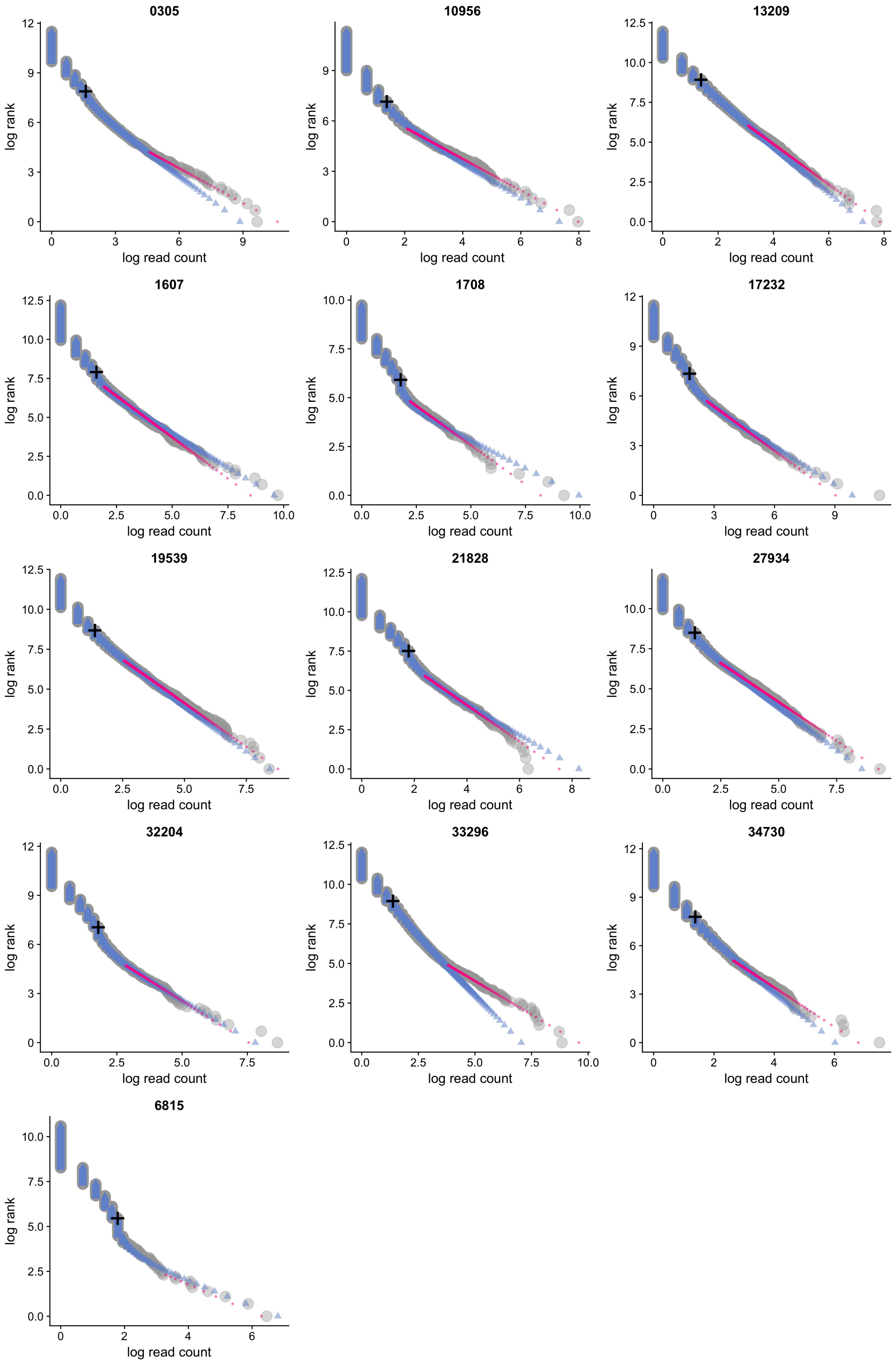

Figure C: Plots of empirical glioma data and the fitted curves based on the Desponds method (pink) and our method (blue). The cross marks the threshold estimated by our method.

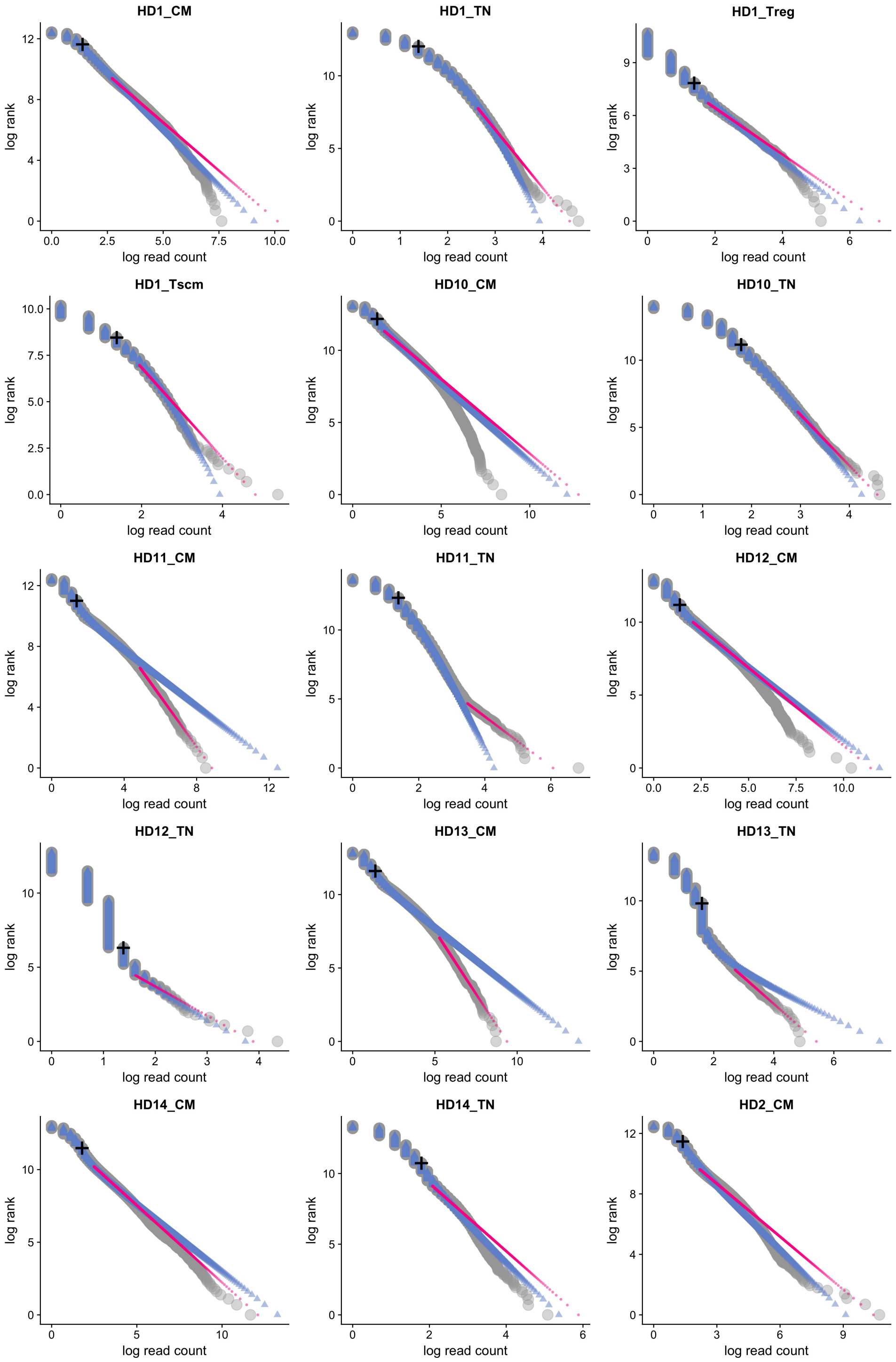
HD1_CM
HD1_TN
HD1_Treg
HD1_Tscm
HD10_CM
HD10_TN
HD11_CM
HD11_TN
HD12_CM
HD12_TN
HD13_CM
HD13_TN
HD14_CM
HD14_TN
HD2_CM
log rank
log read count

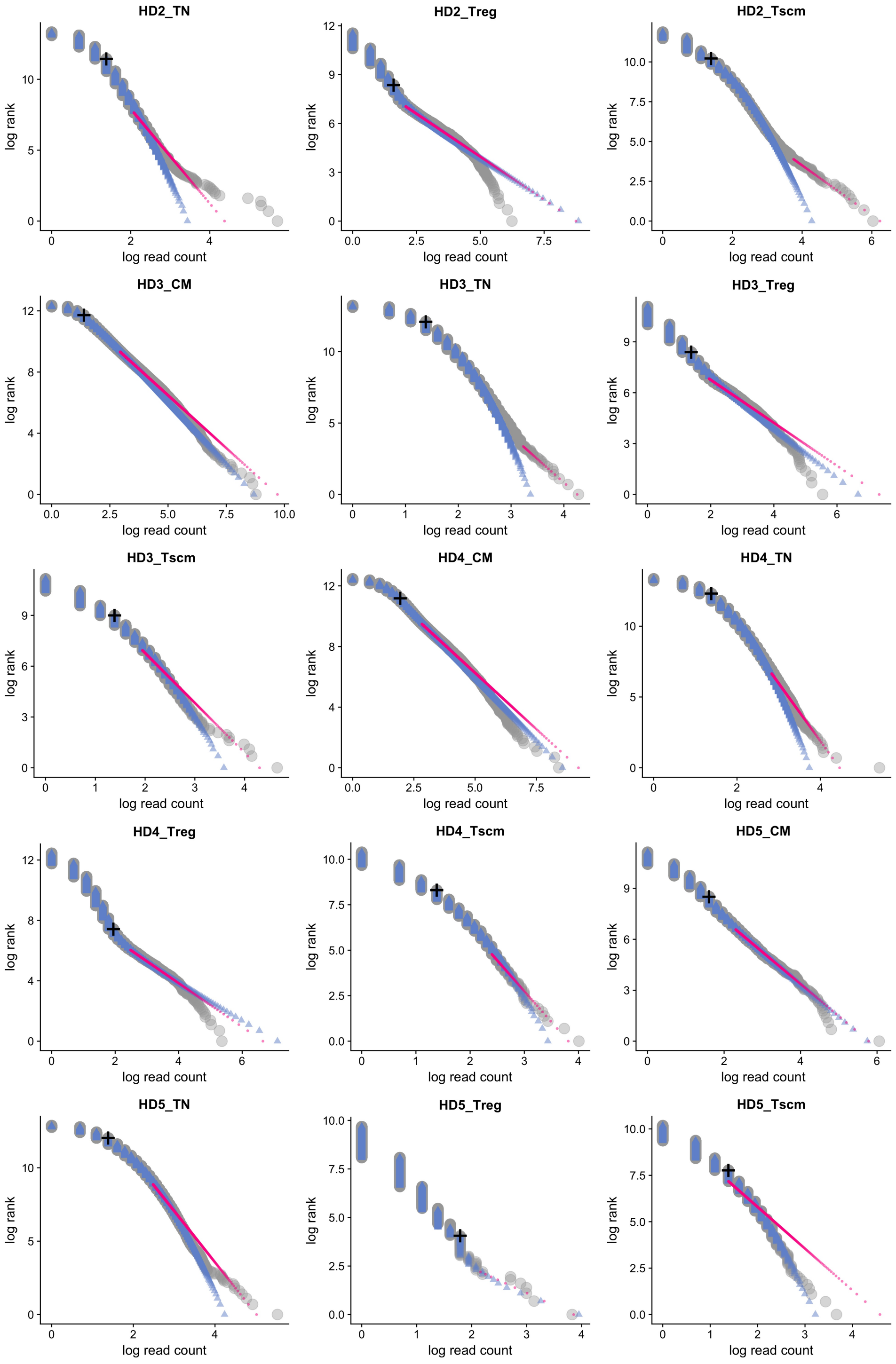
HD2_TN
HD2_Treg
HD2_Tscm
HD3_CM
HD3_TN
HD3_Treg
HD3_Tscm
HD4_CM
HD4_TN
HD4_Treg
HD4_Tscm
HD5_CM
HD5_TN
HD5_Treg
HD5_Tscm
log rank
log read count

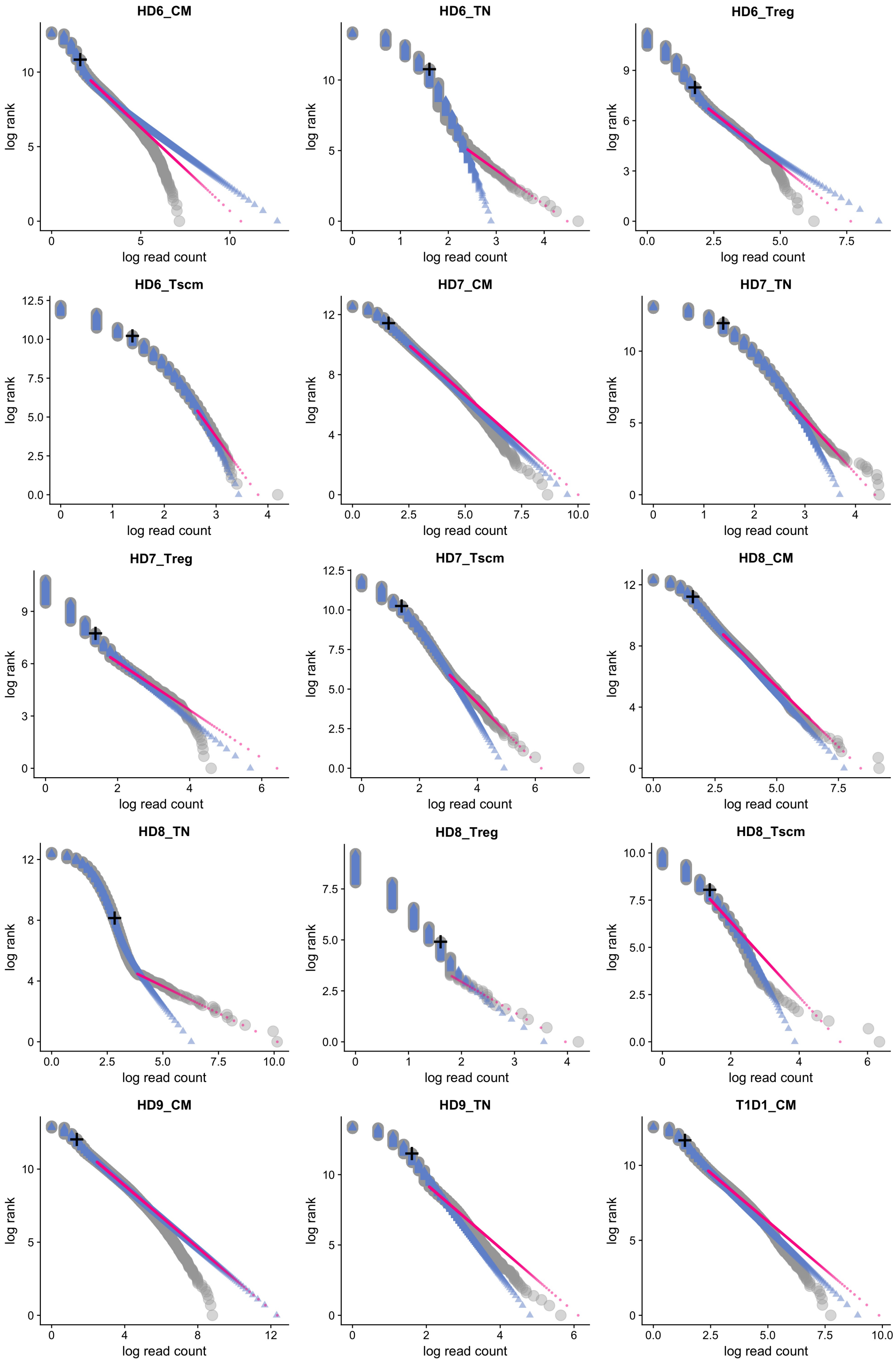
HD6_CM
HD6_TN
HD6_Treg
HD6_Tscm
HD7_CM
HD7_TN
HD7_Treg
HD7_Tscm
HD8_CM
HD8_TN
HD8_Treg
HD8_Tscm
HD9_CM
HD9_TN
T1D1_CM
log rank
log read count

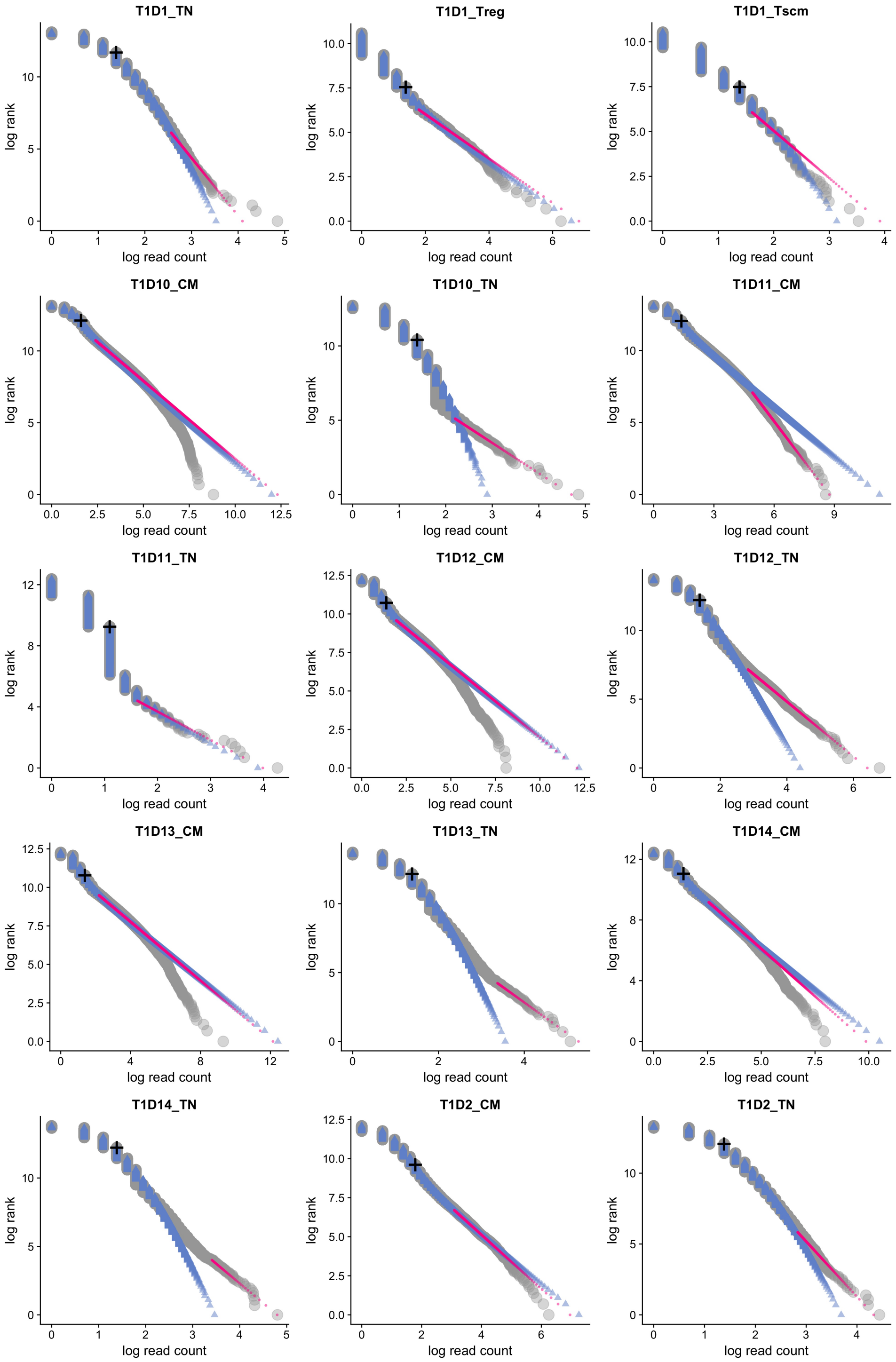
T1D1_TN
T1D1_Treg
T1D1_Tscm
T1D10_CM
T1D10_TN
T1D11_CM
T1D11_TN
T1D12_CM
T1D12_TN
T1D13_CM
T1D13_TN
T1D14_CM
T1D14_TN
T1D2_CM
T1D2_TN
log rank
log read count

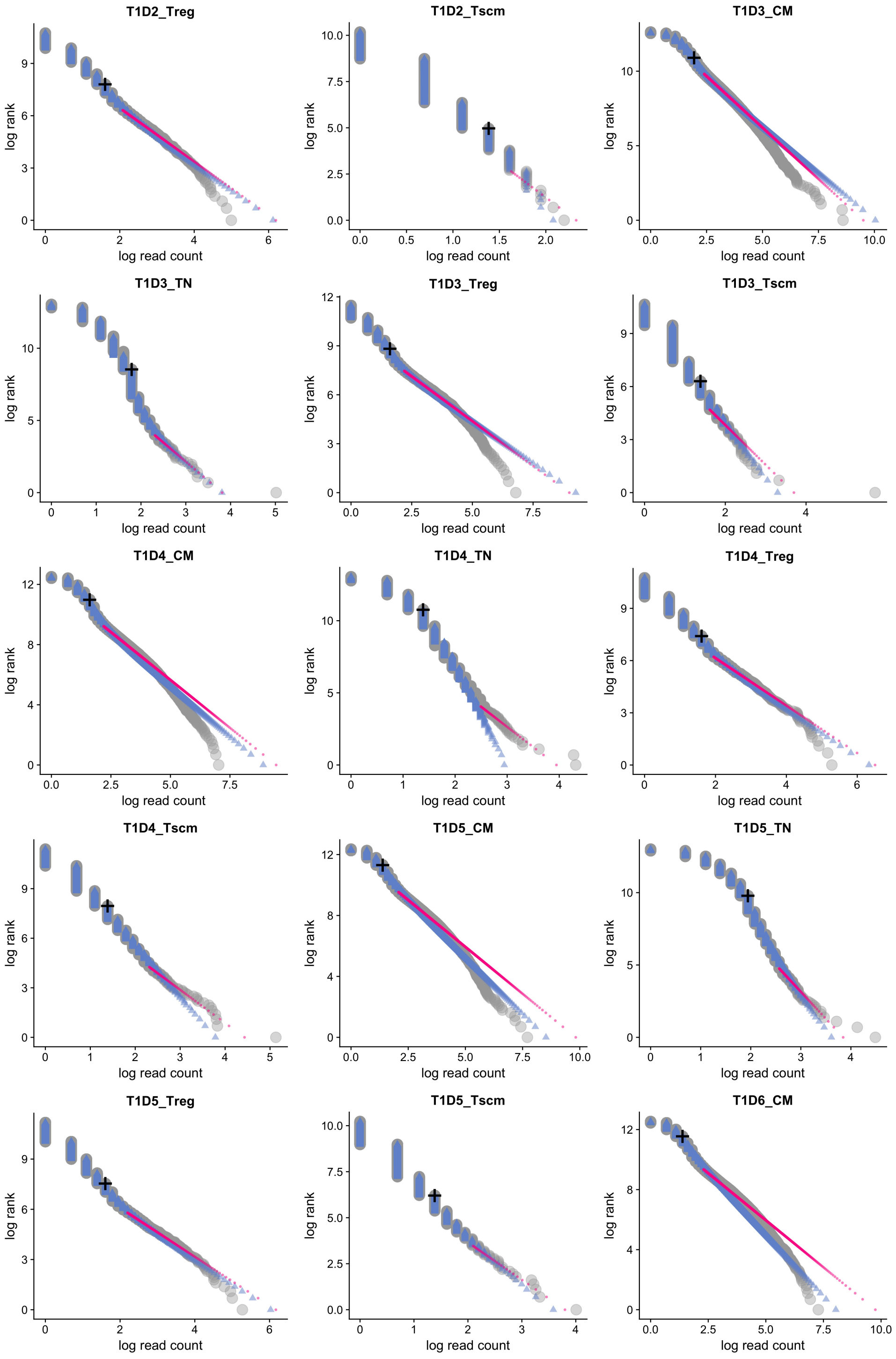
T1D2_Treg
T1D2_Tscm
T1D3_CM
T1D3_TN
T1D3_Treg
T1D3_Tscm
T1D4_CM
T1D4_TN
T1D4_Treg
T1D4_Tscm
T1D5_CM
T1D5_TN
T1D5_Treg
T1D5_Tscm
T1D6_CM
log rank
log read count

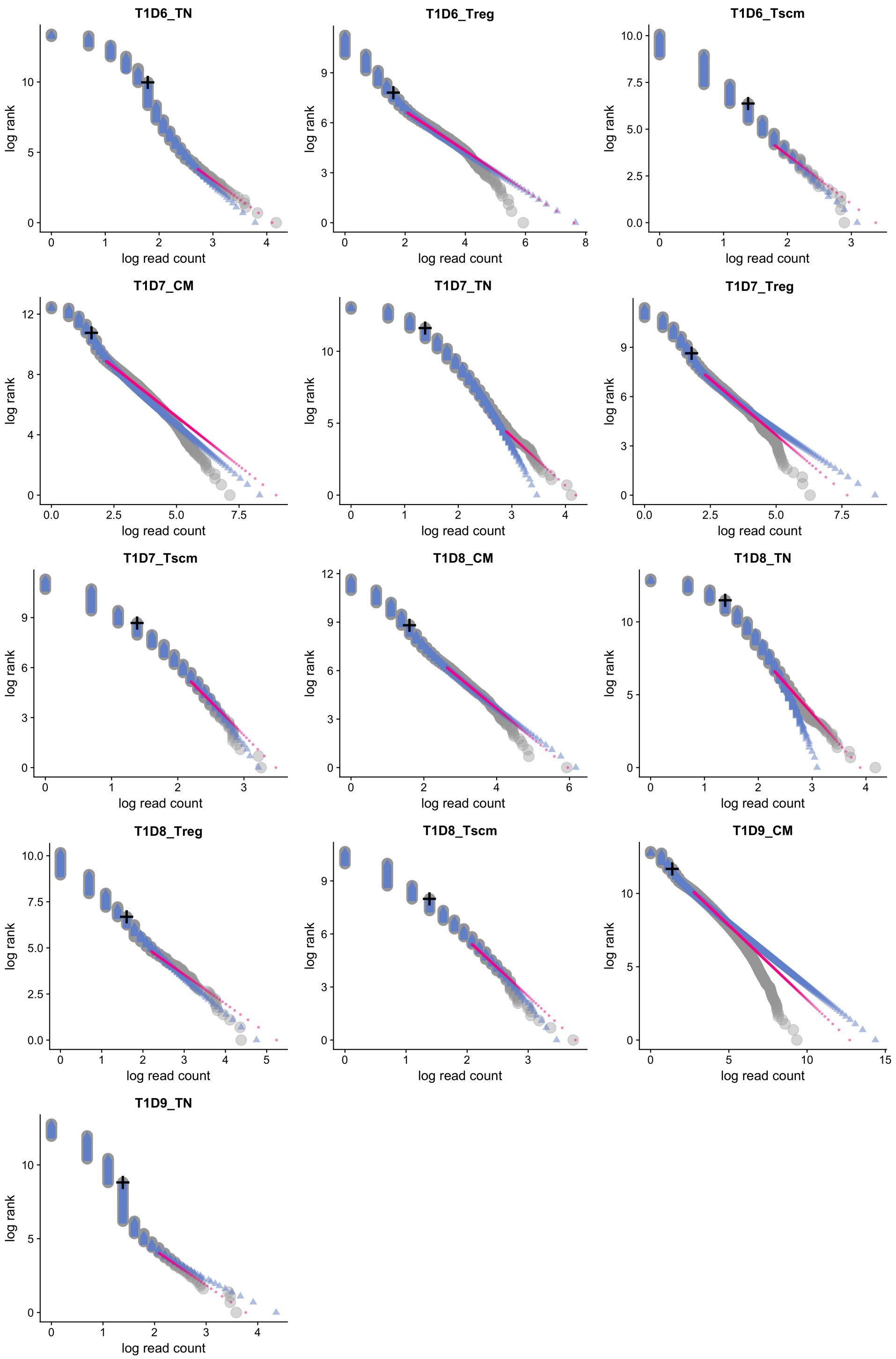

Figure D: Plots of empirical type 1 diabetes versus healthy donor data and the fitted curves based on the Desponds method (pink) and our method (blue). The cross marks the threshold estimated by our method.
