# Supplementary material for: powerTCR: A model-based approach to comparative analysis of the clone size distribution of the T cell receptor repertoire
Source: PLoS Comput Biol. 2018 Nov 28;14(11):e1006571. doi: 10.1371/journal.pcbi.1006571 (PMC6287877; doi:10.1371/journal.pcbi.1006571)
Supplement: S2 Text — Details on simulation study comparing the discrete model and Desponds et al. model, and simulation study comparing the discrete and continuous models. (PDF) [file pcbi.1006571.s002.pdf]

Supplementary file 2 — Simulation for comparing the accuracy of parameter estimation for the discrete spliced threshold model, the Desponds et al. model, and the continuous spliced threshold model

Algorithm 1 Simulate data from discrete Gamma-GPD spliced threshold model

define $Q^{(G)}_{\alpha,\beta,u}$:= quantile function of a discrete truncated Gamma distribution with shape and rate α and β, respectively, and truncation point u
define $Q^{(GPD)}_{u,\sigma,\xi}$:= quantile function of a discrete GPD with threshold, scale, and shape u, σ, and ξ, respectively
Simulate $w \sim$ Unif$(0, 1)$
if $w \geq 1 - \phi$, **then**
    $w' \leftarrow [w - (1 - \phi)]/\phi$.
    Compute $Q^{(GPD)}_{\alpha,\beta,u}(w')$
else
    $w' \leftarrow w/[1 - \phi]$
    Compute $Q^{(G)}_{u,\sigma,\xi}(w')$

Algorithm 2 Simulate data from continuous Gamma-GPD spliced threshold model

define $Q^{(G)}_{\alpha,\beta,u}$:= quantile function of a truncated Gamma distribution with shape and rate α and β, respectively, and truncation point u
define $Q^{(GPD)}_{u,\sigma,\xi}$:= quantile function of a GPD with threshold, scale, and shape u, σ, and ξ, respectively
Simulate $w \sim$ Unif$(0, 1)$
if $w \geq 1 - \phi$, **then**
    $w' \leftarrow [w - (1 - \phi)]/\phi$.
    Compute $Q^{(GPD)}_{\alpha,\beta,u}(w')$
else
    $w' \leftarrow w/[1 - \phi]$
    Compute $Q^{(G)}_{u,\sigma,\xi}(w')$

To compare the performance of the discrete spliced model, the continuous spliced model, and the Desponds et al. model, we performed simulations to evaluate the accuracy of parameter estimation. In an attempt to generate realistic datasets, we first fit our model to multiple real TCR repertoire libraries and obtained plausible parameter settings for simulations. Using the combinations of parameters in the plausible range, we simulated 24 scenarios (Table A), 6 of which are displayed in Figs A and B. The range considered include $\alpha \in \{1, 3, 5, 10\}$, $\xi \in \{.25, .75, 1.1\}$, $\phi \in \{0.1, 0.15\}$, $\beta = 0.15$, $\sigma = \frac{\sqrt{\alpha}}{\beta}$, and $u = \lfloor Q_{\alpha,\beta}(1-\phi) \rfloor$, where $Q_{\alpha,\beta}$ is the quantile function of the Gamma distribution with mean $\frac{\alpha}{\beta}$. For a given set of parameters ϕ, α, β, u, σ, and ξ, data from the discrete and continuous versions of the model are simulated according to Algorithms 1 and 2, respectively.

For each simulation setting, we generated 1,000 replications of the dataset. 95% confidence intervals of the estimates were obtained from the replications. This pro-

Table A: Parameters used in simulation study. Bolded settings are those 6 which are also plotted here.

ϕ	α	β	u	σ	ξ
0.1	1	0.15	15	6.67	0.25
0.1	1	0.15	15	6.67	0.75
0.1	1	0.15	15	6.67	1.1
0.1	3	0.15	35	11.55	0.25
0.1	3	0.15	35	11.55	0.75
0.1	3	0.15	35	11.55	1.1
0.1	5	0.15	53	14.91	0.25
0.1	5	0.15	53	14.91	0.75
0.1	5	0.15	53	14.91	1.1
0.1	10	0.15	94	21.08	0.25
0.1	10	0.15	94	21.08	0.75
0.15	10	0.15	94	21.08	1.1
**0.15**	**1**	**0.15**	**12**	**6.67**	**0.25**
**0.15**	**1**	**0.15**	**12**	**6.67**	**0.75**
**0.15**	**1**	**0.15**	**12**	**6.67**	**1.1**
**0.15**	**3**	**0.15**	**31**	**11.55**	**0.25**
**0.15**	**3**	**0.15**	**31**	**11.55**	**0.75**
**0.15**	**3**	**0.15**	**31**	**11.55**	**1.1**
0.15	5	0.15	48	14.91	0.25
0.15	5	0.15	48	14.91	0.75
0.15	5	0.15	48	14.91	1.1
0.15	10	0.15	88	21.08	0.25
0.15	10	0.15	88	21.08	0.75
0.15	10	0.15	88	21.08	1.1

cedure is employed because the likelihood function of the spliced threshold model is non-differentiable at the threshold which makes the standard maximum likelihood theory inapplicable to its asymptotics. For a detailed explanation, see the review by Carl Scarrott [1]. We thus deem empirical estimates to be more reliable, and in practice recommend a parametric bootstrap to derive confidence bands on real data.

Figs A and B compare the parameters estimated from the discrete spliced threshold model with those from the Despond et al. model and the continuous spliced threshold model, respectively. As the Desponds et al. model only has two parameters, we only compared these two parameters with their counterparts in our model. We examined all 5 parameters when comparing with the continuous spliced threshold model. As shown in Fig A, the Desponds et al. model tends to overestimate the true threshold, especially for larger values of ξ. Estimates also are less stable, that is, have much looser confidence bands, than those from the discrete spliced threshold model. As shown in Fig B, estimation of model parameters is improved when employing the discretized spliced threshold model over its continuous analog.

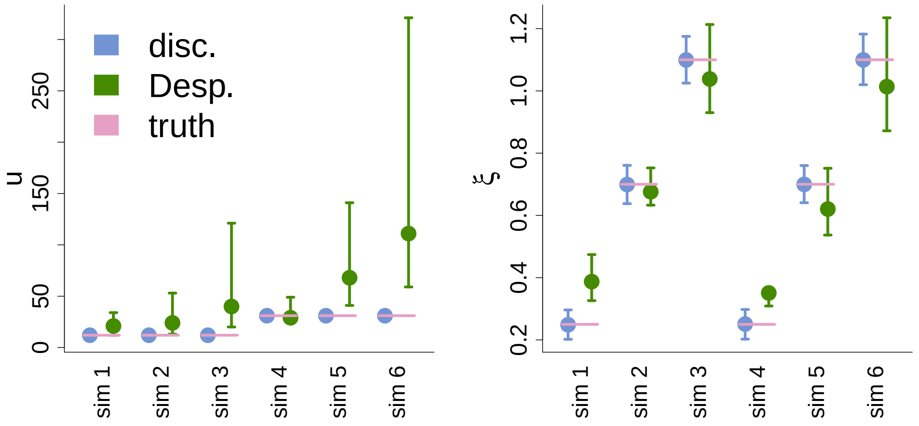

Figure A: Comparison of parameter estimates for the discrete spliced model versus the Desponds et al. model under 6 different simulation scenarios of size $n = 20,000$ clonotypes. Median parameter estimates with 95% confidence intervals, derived from 1,000 replications of each simulation, are plotted against the true parameter value, denoted by the pink horizontal line. To ensure that comparable estimates are being plotted, the tail shape parameter α_d estimated from the Desponds model is displayed as α_d^{-1}. This is done since the shape parameter of the Type-I Pareto distribution is inversely related to the shape parameter of the GPD. Confidence bands on the threshold for the discrete spliced model appear absent, due to the scale of the y-axis.

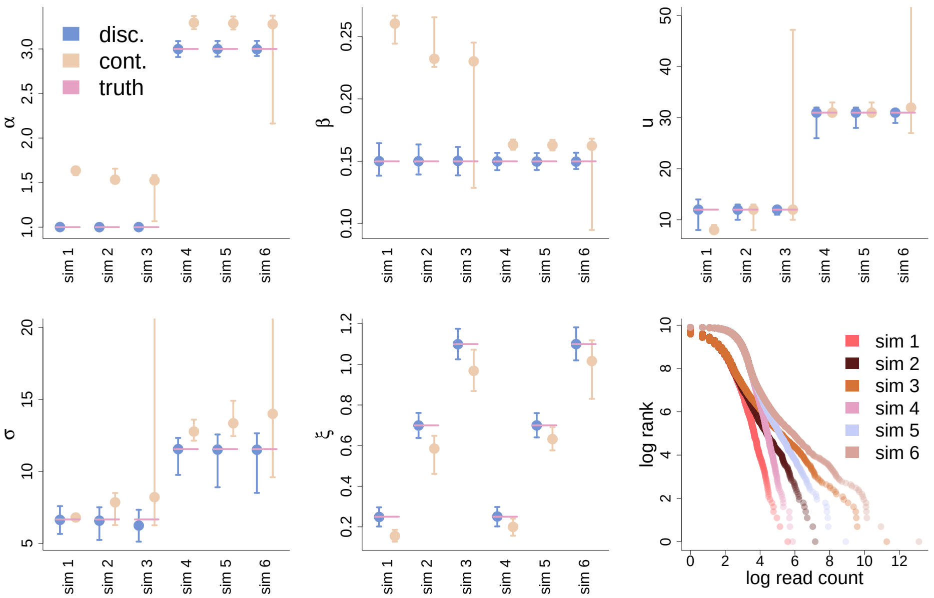

Figure B: Comparison of parameter estimates between the continuous and discrete spliced threshold model for the same datasets as in Fig A. Median parameter estimates with 95% confidence intervals, derived from 1,000 replications of each simulation, are plotted against the true parameter value, denoted by the pink horizontal line. In order to keep margins of the plots in a reasonable range for viewing, the upper bounds of some 95% confidence bands are omitted. Sample simulated data are displayed in the bottom-right panel, plotted on a log-log scale.

References

1. Scarrott C. Univariate Extreme Value Mixture Modeling. In: Dey DK, Yan J, editors. Extreme Value Modeling and Risk Analysis: Methods and Applications. Boca Raton: CRC Press;. p. 41–67.
