# Supplementary material for: powerTCR: A model-based approach to comparative analysis of the clone size distribution of the T cell receptor repertoire
Source: PLoS Comput Biol. 2018 Nov 28;14(11):e1006571. doi: 10.1371/journal.pcbi.1006571 (PMC6287877; doi:10.1371/journal.pcbi.1006571)
Supplement: S3 Text — Information on simulation study that finds strong positive relationship between ξ and the clonality estimator. (PDF) [file pcbi.1006571.s003.pdf]

Supplementary file 3 — Correlation between ξ and clonality

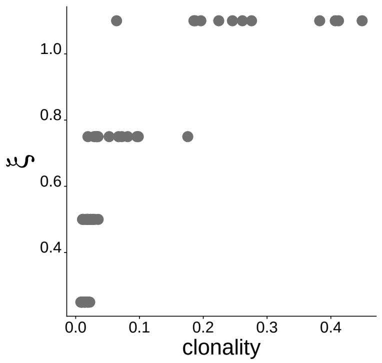

Figure A: Scatterplot of clonality versus ξ on 48 simulated datasets, described in *Correlation between ξ and clonality* in the Methods section.

Table A: Estimates of clonality compared against ξ for 48 simulated samples described in *Correlation between ξ and clonality* in the Methods section.

ξ	clonality											
0.25	0.0183	0.0188	0.0197	0.0220	0.0127	0.0141	0.0143	0.0150	0.0080	0.0090	0.0097	0.0097
0.50	0.0234	0.0271	0.0295	0.0352	0.0173	0.0191	0.0230	0.0264	0.0107	0.0121	0.0190	0.0156
0.75	0.0350	0.0960	0.0816	0.0672	0.0337	0.0723	0.1757	0.0981	0.0192	0.0290	0.0319	0.0524
1.10	0.1874	0.2757	0.3825	0.2613	0.2457	0.1962	0.2242	0.4069	0.0641	0.1853	0.4120	0.4490
