# Supplementary material for: powerTCR: A model-based approach to comparative analysis of the clone size distribution of the T cell receptor repertoire
Source: PLoS Comput Biol. 2018 Nov 28;14(11):e1006571. doi: 10.1371/journal.pcbi.1006571 (PMC6287877; doi:10.1371/journal.pcbi.1006571)
Supplement: S4 Text — Tables summarizing number of unique clonotypes per sample, total reads sequenced per sample, and other patient-specific information for the Sarcoidosis and glioblastoma datasets. Additionally, tables containing fitted model parameter estimates for both our model and the competing model, as well as ecological estimator values, computed for every sample. (PDF) [file pcbi.1006571.s004.pdf]

Supplementary file 4 — Summaries for the four TCR repertoire datasets

Table A: Summary of unique clonotypes sequences from the MHC-II-positive and control groups of mice in the breast cancer study.

ID	group	unique clonotypes	total reads
C54	control	4200	383783
C55	control	4385	255654
C56	control	6751	432783
M62	treatment	11240	575313
M63	treatment	10044	509123
M64	treatment	4932	528723

Table B: Estimated parameter values for the mouse tumor data described in *Discrimination between treatment and control in tumor samples* with 95% confidence intervals derived from 1,000 bootstrap replicates. Bootstrapping was done in the manner described by [1]. Briefly, resampling is performed separately above and below the estimated threshold in order to preserve $\hat{\phi}$ with respect to the inital threshold estimate.

	ϕ		α		β		u		σ		ξ	
	est.	95% CI	est.	95% CI	est.	95% CI	est.	95% CI	est.	95% CI	est.	95% CI
C54	0.206	(.186, .225)	0.349	(.330, .367)	0.017	(.014, .021)	65	(57, 75)	130.5	(107.1, 157.5)	0.592	(.463, .712)
C55	0.246	(.209, .246)	0.529	(.496, .562)	0.042	(.033, .048)	38	(38, 48)	59.81	(53.89, 74.19)	0.610	(.482, .712)
C56	0.250	(.217, .250)	0.512	(.484, .535)	0.048	(.041, .055)	31	(31, 39)	48.16	(44.37, 56.56)	0.736	(.653, .837)
M62	0.241	(.215, .250)	0.618	(.595, .644)	0.074	(.066, .081)	25	(24, 29)	28.26	(26.32, 30.61)	0.802	(.728, .872)
M63	0.215	(.193, .243)	0.623	(.593, .646)	0.083	(.070, .090)	24	(23, 31)	26.76	(24.85, 29.71)	0.821	(.747, .913
M64	0.148	(.121, .230)	0.647	(.611, .711)	0.109	(.093, .141)	26	(16, 33)	22.59	(16.04, 25.55)	1.187	(.992, 1.40)

Table C: The thresholds and shape parameters inferred with the Desponds et al. model and the ecological estimators computed on the mouse tumor data. Additionally, the proportion of highly stimulated clones, as computed from the spliced model.

	C54	C55	C56	M62	M63	M64
Desponds u	357	112	219	164	33	11
Desponds α_d	1.50	1.33	1.06	0.99	1.08	0.89
richness	4200	4385	6751	11240	10044	4932
Shannon	6.37	6.45	6.33	6.41	6.02	3.17
clonality	0.24	0.23	0.28	0.31	0.35	0.63
prop. highly stimulated	0.885	0.872	0.903	0.892	0.895	0.948

Table D: Summary of unique clonotypes sequences from LS and non-LS Sarcoidosis patients.

patient ID	group	unique clonotypes	total reads
1000	non-LS	20804	594996
1025	non-LS	358	93838
1127	non-LS	987	227999
1180	non-LS	16834	894789
1079	LS	17658	3341997
1088	LS	3557	326015
1102	LS	21503	3545267
1124	LS	10317	1742161
1146	LS	6843	780544
1147	LS	5337	643873
1162	LS	1080	210842
1177	LS	4827	761934
1215	LS	6674	502308

Table E: Estimated parameter values for the LS data described in Differentiation between Sarcoidosis patients with 95% confidence intervals derived from 1,000 bootstrap replicates.

	ϕ		α		β		u		σ		ξ	
ID	est.	95% CI	est.	95% CI	est.	95% CI	est.	95% CI	est.	95% CI	est.	95% CI
1000	0.073	(0.059,0.010)	0.837	(0.819,0.865)	0.042	(0.040,0.045)	69	(59,77)	28.9	(25.4,31.4)	0.755	(0.639,0.874)
1025	0.120	(0.101,0.282)	0.165	(0.135,0.200)	1.6×10^{-7}	$(2.3\times10^{-11}, 1.9\times10^{-7})$	614	(255,700)	844	(338, 1389)	-0.066	(-0.402,0.468)
1127	0.250	(0.246,0.250)	0.072	(0.062,0.082)	2.3×10^{-9}	$(1.1\times10^{-10},4.6\times10^{-7})$	166	(166,176)	416	(330,513)	0.422	(0.247,0.607)
1180	0.193	(0.177,0.256)	0.612	(0.593,0.634)	0.053	(0.048,0.060)	35	(25,39)	40.9	(30.1,44.7)	0.986	(0.915,1.05)
1079	0.178	(0.170,0.189)	0.363	(0.354,0.375)	0.013	(0.012,0.014)	128	(118,138)	277	(233,313)	0.803	(0.734,0.890)
1088	0.190	(0.179,0.203)	0.310	(0.296,0.324)	3.7×10^{-8}	$(3.4\times10^{-10},1.9\times10^{-6})$	129	(120,135)	147	(131,170)	0.328	(0.223,0.415)
1102	0.251	(0.239,0.251)	0.255	(0.250,0.260)	2.0×10^{-8}	$(5.2\times10^{-10},1.6\times10^{-6})$	89	(89,95)	144	(137,155)	0.856	(0.807,0.899)
1124	0.241	(0.229,0.248)	0.233	(0.227,0.240)	8.9×10^{-9}	$(1.7\times10^{-10},8.9\times10^{-7})$	134	(127,144)	243	(225,263)	0.508	(0.451,0.564)
1146	0.241	(0.221,0.250)	0.259	(0.250,0.269)	1.7×10^{-6}	$(3.8\times10^{-10},2.3\times10^{-6})$	89	(84,99)	155	(141,178)	0.568	(0.483,0.631)
1147	0.244	(0.226,0.250)	0.341	(0.324,0.362)	0.001	$(1.2\times10^{-7},0.005)$	81	(79,91)	94.7	(85.9,105)	0.666	(0.563,0.774)
1162	0.199	(0.194,0.203)	0.203	(0.184,0.224)	2.7×10^{-9}	$(4.8\times10^{-11},2.4\times10^{-7})$	267	(257,271)	518	(439,619)	-0.072	(-0.215,0.003)
1177	0.183	(0.177,0.191)	0.244	(0.235,0.255)	9.5×10^{-8}	$(2.7\times10^{-10},1.3\times10^{-6})$	212	(202,218)	247	(222,280)	0.482	(0.384,0.565)
1215	0.250	(0.219,0.250)	0.422	(0.402,0.445)	0.004	(0.402,0.445)	72	(72,82)	81.8	(77.0,94.0)	0.559	(0.483, 0.619)

Table F: The thresholds and shape parameters inferred with the Desponds et al. model and the ecological estimators computed on the Sarcoidosis patient data. Additionally, the proportion of highly stimulated clones, as computed from the spliced model.

	1000	1025	1127	1180	1079	1088	1102	1124	1146	1147	1162	1177	1215
Desponds u	48	135	728	31	2038	459	251	691	880	124	868	381	803
Desponds α_d	1.74	0.86	1.68	0.90	1.56	2.23	1.00	1.54	1.79	1.22	2.93	1.45	2.17
richness	20804	358	987	16834	17658	3557	21503	10317	6843	5337	1080	4827	6674
Shannon	8.97	4.74	5.24	7.49	7.65	7.20	8.08	7.78	7.38	6.26	5.95	7.21	7.68
clonality	0.10	0.19	0.24	0.23	0.22	0.12	0.19	0.16	0.16	0.27	0.15	0.15	0.13
prop. highly stimulated	0.435	0.644	0.943	0.867	0.908	0.709	0.910	0.870	0.868	0.874	0.765	0.771	0.801

Table G: Summary of clinical outcome and unique clonotypes sequences from glioblastoma patients, sorted by TTP. Estimated TIL count and tumor/PBMC overlap were computed in [2]

patient ID	TTP	OS	estimated TIL count	tumor/PBMC overlap	unique clonotypes	total reads
10956	0.5	11.4	0.142986	5.6%	82441	110155
6815	0.9	4.5	0.543669	10.5%	39783	48643
33296	1.2	8.1	0.021454	0.2%	164657	288433
34730	2.3	5.2	0.087014	3.1%	129154	164348
19539	2.6	30	0.112551	8.2%	148650	259143
21828	3.9	6.3	0.492902	4.9%	179366	229211
1708	8.0	28.7	0.787013	64.2%	16939	45596
1607	9.7	20.7	0.177036	24.2%	197759	301230
32204	12.6	12.6	0.033937	0.7%	111273	155581
0305	15.3	23.5	0.414797	24.9%	98448	227474
13209	45.1	48.3	1.096282	0.5%	154587	253184
17232	57.6	71.3	2.527273	66.1%	96062	238949
27934	85.8	85.8	1.168437	21.1%	141589	244805

Table H: Estimated parameter values and 95% confidence intervals, generated from 1,000 bootstrap replicates, for the glioblastoma PBMC data described in Relationship between the landscape of the clone size distribution and clinical outcome. Patients are sorted by TTP.

	ϕ		α		β		u		σ		ξ	
ID	est.	95% CI	est.	95% CI	est.	95% CI	est.	95% CI	est.	95% CI	est.	95% CI
10956	0.016	(0.010,0.016)	0.350	(0.295,0.379)	1.10	(0.938,1.17)	4	(4,5)	1.54	(1.40,2.01)	0.957	(0.853,1.24)
6815	0.006	(0.006,0.006)	0.170	(0.157,0.184)	0.525	(0.479,0.569)	6	(6,6)	0.448	(0.293,0.700)	1.46	(1.08,1.78)
33296	0.047	(0.030,0.047)	0.442	(0.390,0.462)	0.825	(0.702,0.866)	4	(4,5)	1.79	(1.72,2.16)	0.679	(0.636,0.790)
34730	0.019	(0.019,0.019)	0.444	(0.419,0.471)	1.157	(1.10,1.22)	4	(4,4)	1.63	(1.51,1.76)	0.655	(0.582,0.730)
19539	0.040	(0.040,0.040)	0.428	(0.406,0.449)	0.917	(0.867,0.961)	4	(4,4)	2.12	(2.02,2.22)	0.870	(0.820,0.923)
21828	0.010	(0.010,0.017)	0.139	(0.134,0.162)	0.435	(0.416,0.533)	6	(5,6)	1.42	(1.28,1.56)	1.06	(0.749,1.15)
1708	0.022	(0.022,0.022)	0.261	(0.239,0.285)	0.467	(0.417,0.520)	6	(6,6)	0.970	(0.719,1.32)	1.78	(1.51,2.03)
1607	0.014	(0.014,0.014)	0.232	(0.224,0.242)	0.715	(0.689,0.742)	5	(5,5)	1.65	(1.51,1.80)	1.17	(1.08,1.25)
32204	0.010	(0.010,0.010)	0.194	(0.186,0.202)	0.471	(0.449,0.493)	6	(6,6)	1.12	(0.987,1.27)	1.10	(0.976,1.23)
0305	0.027	(0.019,0.027)	0.300	(0.270,0.314)	0.628	(0.549,0.659)	5	(5,6)	2.04	(1.90,2.52)	1.04	(0.970,1.28)
13209	0.049	(0.049,0.049)	0.449	(0.430,0.467)	0.867	(0.829,0.905)	4	(4,4)	2.11	(2.03,2.19)	0.683	(0.644,0.725)
17232	0.016	(0.016,0.016)	0.194	(0.186,0.202)	0.416	(0.394,0.441)	6	(6,6)	1.43	(1.28,1.62)	1.33	(1.21,1.44)
27934	0.035	(0.023,0.035)	0.397	(0.332,0.418)	0.946	(0.775,0.996)	4	(4,5)	1.90	(1.81,2.27)	0.925	(0.872,1.10)

Table I: The thresholds and shape parameters inferred with the Desponds et al. model and the ecological estimators computed on the glioblastoma PBMC data. Additionally, the proportion of highly stimulated clones, as computed from the spliced model.

	0305	10956	13209	1607	1708	17232	19539	21828	27934	32204	33296	34730	6815
Desponds u	98	8	22	7	9	14	13	11	12	17	45	14	24
Desponds α_d	0.67	0.94	1.27	1.06	0.80	0.89	1.09	1.16	0.97	1.00	0.848	1.22	0.77
richness	82441	39783	164657	129154	148650	179366	16939	197759	111273	98448	154587	96062	141589
Shannon	10.62	10.34	10.82	11.45	10.76	11.77	6.59	10.80	10.91	8.76	11.26	7.54	10.49
clonality	0.06	0.02	0.10	0.03	0.10	0.03	0.32	0.11	0.06	0.24	0.06	0.34	0.12
prop. highly stimulated	0.190	0.067	0.351	0.134	0.359	0.113	0.540	0.269	0.157	0.494	0.312	0.519	0.362

Table J: Summary of unique clonotypes sequences from healthy donors and individuals with T1D.

ID	group	unique clonotypes	total reads
HD1_CM	HD	250236	1498268
HD1_TN	HD	440905	1555345
HD1_Treg	HD	42987	74551
HD1_Tscm	HD	26163	64670
HD10_CM	HD	488176	3336371
HD10_TN	HD	1276177	3639099
HD11_CM	HD	250109	1244428
HD11_TN	HD	878903	2564403
HD12_CM	HD	421593	1698036
HD12_TN	HD	337679	447845
HD13_CM	HD	386343	2504005
HD13_TN	HD	683043	1376854
HD14_CM	HD	444150	3429897
HD14_TN	HD	637046	1878512
HD2_CM	HD	266244	1352190
HD2_TN	HD	610156	1485758
HD2_Treg	HD	103075	204154
HD2_Tscm	HD	142717	380388
HD3_CM	HD	230115	1646129
HD3_TN	HD	555941	1762742
HD3_Treg	HD	64726	118347
HD3_Tscm	HD	69621	138954
HD4_CM	HD	257561	1764632
HD4_TN	HD	620855	2076350
HD4_Treg	HD	253386	481107
HD4_Tscm	HD	32337	66275
HD5_CM	HD	67717	148292
HD5_TN	HD	389324	1526049
HD5_Treg	HD	15936	20446
HD5_Tscm	HD	26262	48174
HD6_CM	HD	319165	1291649
HD6_TN	HD	649422	1654707
HD6_Treg	HD	74227	163592
HD6_Tscm	HD	191030	424572
HD7_CM	HD	292927	1814535
HD7_TN	HD	519906	1601446
HD7_Treg	HD	48104	76103
HD7_Tscm	HD	152471	393173
HD8_CM	HD	233334	1194356
HD8_TN	HD	252570	1282098
HD8_Treg	HD	10105	13980
HD8_Tscm	HD	22158	49381
HD9_CM	HD	409677	3197281
HD9_TN	HD	682897	2076711

ID	group	unique clonotypes	total reads
T1D1_CM	T1D	297671	1475536
T1D1_TN	T1D	486446	1368764
T1D1_Treg	T1D	39331	65348
T1D1_Tscm	T1D	37705	61837
T1D10_CM	T1D	515054	3911851
T1D10_TN	T1D	331364	751438
T1D11_CM	T1D	493471	2717824
T1D11_TN	T1D	235542	327725
T1D12_CM	T1D	211711	1007301
T1D12_TN	T1D	906973	2526515
T1D13_CM	T1D	211660	1126106
T1D13_TN	T1D	906527	2441013
T1D14_CM	T1D	253210	1090840
T1D14_TN	T1D	986439	2639172
T1D2_CM	T1D	178423	528610
T1D2_TN	T1D	617242	1848922
T1D2_Treg	T1D	46142	90158
T1D2_Tscm	T1D	26235	33230
T1D3_CM	T1D	303193	1687191
T1D3_TN	T1D	457704	1039497
T1D3_Treg	T1D	95954	235156
T1D3_Tscm	T1D	41933	57833
T1D4_CM	T1D	269762	1099257
T1D4_TN	T1D	462254	1020918
T1D4_Treg	T1D	46762	80865
T1D4_Tscm	T1D	88503	134064
T1D5_CM	T1D	231629	1025817
T1D5_TN	T1D	444034	1420377
T1D5_Treg	T1D	71850	115377
T1D5_Tscm	T1D	27609	38068
T1D6_CM	T1D	277784	1231045
T1D6_TN	T1D	648213	1732265
T1D6_Treg	T1D	76123	133248
T1D6_Tscm	T1D	23532	34282
T1D7_CM	T1D	263093	957336
T1D7_TN	T1D	482536	1350134
T1D7_Treg	T1D	89188	234359
T1D7_Tscm	T1D	80487	149962
T1D8_CM	T1D	114290	239088
T1D8_TN	T1D	407562	1139023
T1D8_Treg	T1D	25755	41054
T1D8_Tscm	T1D	42034	76739
T1D9_CM	T1D	377076	2937682
T1D9_TN	T1D	345571	542873

Table K: Estimated parameter values and 95% confidence intervals, generated from 1,000 bootstrap replicates, for the healthy donors in the data described in Relationships among sorted CD4$^+$ T cell subtypes in individuals with type 1 diabetes and healthy donors.

	ϕ		α		β		u		σ		ξ	
ID	est.	95% CI	est.	95% CI	est.	95% CI	est.	95% CI	est.	95% CI	est.	95% CI
HD1_CM	0.451	(0.451,0.451)	3.529	(3.470,3.588)	1.667	(1.623,1.707)	4	(4, 4)	2.395	(2.37,2.42)	0.670	(0.661,0.680)
HD1_TN	0.377	(0.377,0.377)	2.866	(2.831,2.900)	1.299	(1.273,1.323)	4	(4, 4)	2.091	(2.08,2.11)	0.096	(0.090,0.102)
HD1_Treg	0.060	(0.039,0.06)	0.880	(0.786,0.941)	1.152	(1.007,1.245)	4	(4, 5)	1.906	(1.75,2.29)	0.666	(0.609,0.801)
HD1_Tscm	0.180	(0.180,0.180)	1.638	(1.557,1.739)	1.310	(1.227,1.407)	4	(4, 4)	2.325	(2.22,2.44)	0.187	(0.142,0.230)
HD10_CM	0.402	(0.402,0.402)	4.960	(4.915,5.005)	2.673	(2.642,2.703)	4	(4, 4)	2.444	(2.42,2.46)	0.912	(0.904,0.919)
HD10_TN	0.055	(0.055,0.055)	2.477	(2.467,2.486)	1.077	(1.071,1.082)	6	(6, 6)	1.704	(1.68,1.73)	0.188	(0.178,0.197)
HD11_CM	0.243	(0.243,0.243)	6.250	(6.185,6.311)	3.822	(3.776,3.863)	4	(4, 4)	1.734	(1.71,1.76)	1.089	(1.075,1.104)
HD11_TN	0.258	(0.258,0.258)	3.561	(3.536,3.586)	1.900	(1.881,1.917)	4	(4, 4)	1.441	(1.43,1.45)	0.185	(0.180,0.190)
HD12_CM	0.173	(0.173,0.173)	3.636	(3.603,3.668)	2.558	(2.530,2.583)	4	(4, 4)	2.002	(1.97,2.03)	1.000	(0.987,1.014)
HD12_TN	0.002	(0.002,0.039)	1.919	(1.888,11.376)	2.405	(2.369,13.364)	4	(3, 4)	0.628	(0.11,0.76)	0.566	(0.418,0.694)
HD13_CM	0.285	(0.285,0.285)	5.922	(5.872,5.969)	3.631	(3.598,3.664)	4	(4, 4)	2.194	(2.17,2.22)	1.125	(1.115,1.137)
HD13_TN	0.027	(0.027,0.027)	2.944	(2.927,2.959)	2.010	(1.997,2.022)	5	(5, 5)	0.158	(0.15,0.17)	0.947	(0.909,0.985)
HD14_CM	0.220	(0.220,0.307)	1.999	(1.989,2.307)	0.760	(0.753,0.998)	6	(5, 6)	2.420	(2.24,2.44)	1.084	(0.911,1.096)
HD14_TN	0.072	(0.072,0.072)	2.895	(2.879,2.910)	1.302	(1.294,1.310)	6	(6, 6)	1.421	(1.40,1.44)	0.376	(0.364,0.387)
HD2_CM	0.361	(0.361,0.361)	4.853	(4.789,4.917)	2.524	(2.482,2.567)	4	(4, 4)	1.716	(1.70,1.73)	0.719	(0.707,0.730)
HD2_TN	0.153	(0.153,0.153)	4.073	(4.042,4.102)	2.429	(2.408,2.452)	4	(4, 4)	1.040	(1.03,1.05)	0.133	(0.122,0.143)
HD2_Treg	0.042	(0.027,0.042)	1.100	(1.019,1.123)	1.088	(0.986,1.112)	5	(5, 6)	1.577	(1.50,1.77)	1.004	(0.956,1.31)
HD2_Tscm	0.193	(0.193,0.193)	3.285	(3.234,3.345)	2.488	(2.442,2.537)	4	(4, 4)	2.413	(2.37,2.46)	0.174	(0.157,0.190)
HD3_CM	0.536	(0.536,0.536)	4.040	(3.969,4.117)	1.903	(1.856,1.954)	4	(4, 4)	3.034	(3.01,3.06)	0.603	(0.594,0.612)
HD3_TN	0.325	(0.325,0.325)	4.188	(4.149,4.227)	2.046	(2.020,2.073)	4	(4, 4)	1.560	(1.55,1.57)	0.047	(0.041,0.053)
HD3_Treg	0.068	(0.042,0.068)	1.051	(0.943,1.092)	1.191	(1.042,1.245)	4	(4, 5)	1.593	(1.51,1.97)	0.695	(0.654,0.846)
HD3_Tscm	0.116	(0.116,0.116)	1.744	(1.690,1.800)	1.579	(1.521,1.639)	4	(4, 4)	1.649	(1.59,1.71)	0.158	(0.126,0.190)
HD4_CM	0.281	(0.183,0.281)	2.061	(1.953,2.085)	0.561	(0.500,0.571)	7	(7, 9)	3.885	(3.83,4.54)	0.598	(0.586,0.669)
HD4_TN	0.356	(0.356,0.356)	3.526	(3.491,3.561)	1.718	(1.695,1.743)	4	(4, 4)	1.803	(1.79,1.81)	0.084	(0.079,0.090)
HD4_Treg	0.007	(0.003,0.013)	1.414	(1.399,1.427)	1.077	(1.065,1.088)	7	(6, 9)	2.194	(1.03,5.02)	0.825	(0.652,0.926)
HD4_Tscm	0.125	(0.125,0.125)	1.569	(1.499,1.664)	1.407	(1.333,1.508)	4	(4, 4)	1.891	(1.78,1.98)	0.126	(0.085,0.166)
HD5_CM	0.073	(0.029,0.073)	1.020	(0.987,1.048)	0.733	(0.700,0.761)	5	(5, 7)	1.726	(1.64,3.15)	0.538	(0.448,0.580)
HD5_TN	0.432	(0.432,0.432)	3.607	(3.560,3.653)	1.649	(1.616,1.682)	4	(4, 4)	2.311	(2.29,2.33)	0.128	(0.122,0.133)
HD5_Treg	0.004	(0.004,0.016)	0.874	(0.825,1.121)	1.417	(1.352,1.803)	6	(4, 6)	0.499	(0.20,1.15)	1.152	(0.212,1.647)
HD5_Tscm	0.091	(0.091,0.091)	1.562	(1.477,1.652)	1.510	(1.415,1.615)	4	(4, 4)	1.595	(1.49,1.70)	0.127	(0.075,0.177)
HD6_CM	0.160	(0.160,0.160)	3.545	(3.516,3.575)	1.712	(1.694,1.730)	5	(5, 5)	1.263	(1.23,1.29)	1.160	(1.144,1.179)
HD6_TN	0.074	(0.027,0.074)	4.016	(3.597,4.038)	2.103	(1.805,2.116)	5	(5, 6)	0.850	(0.40,0.86)	0.068	(0.052,0.432)
HD6_Treg	0.040	(0.040,0.066)	1.010	(0.986,1.069)	0.796	(0.775,0.866)	6	(5, 6)	1.752	(1.50,1.91)	1.021	(0.829,1.088)
HD6_Tscm	0.145	(0.145,0.145)	2.904	(2.861,2.946)	2.384	(2.347,2.423)	4	(4, 4)	1.913	(1.88,1.95)	0.064	(0.049,0.077)
HD7_CM	0.319	(0.319,0.319)	3.211	(3.179,3.242)	1.360	(1.341,1.378)	5	(5, 5)	3.251	(3.21,3.29)	0.698	(0.687,0.709)
HD7_TN	0.300	(0.300,0.300)	3.093	(3.064,3.123)	1.508	(1.485,1.529)	4	(4, 4)	1.641	(1.63,1.65)	0.096	(0.089,0.102)
HD7_Treg	0.048	(0.030,0.048)	0.906	(0.799,0.963)	1.247	(1.081,1.326)	4	(4, 5)	1.664	(1.55,1.91)	0.602	(0.543,0.773)
HD7_Tscm	0.187	(0.187,0.187)	2.948	(2.894,2.996)	2.407	(2.360,2.450)	4	(4, 4)	2.065	(2.03,2.10)	0.292	(0.273,0.309)
HD8_CM	0.321	(0.321,0.468)	2.731	(2.701,3.209)	1.004	(0.988,1.383)	5	(4, 5)	2.495	(2.26,2.51)	0.554	(0.489,0.565)
HD8_TN	0.014	(0.009,0.030)	1.641	(1.628,1.676)	0.397	(0.391,0.410)	17	(14, 19)	3.216	(3.01,3.29)	0.551	(0.358,0.730)
HD8_Treg	0.013	(0.006,0.029)	0.846	(0.727,0.987)	1.218	(1.032,1.440)	5	(4, 6)	0.902	(0.28,1.20)	0.635	(0.261,1.64)
HD8_Tscm	0.142	(0.142,0.142)	1.692	(1.602,1.791)	1.435	(1.342,1.538)	4	(4, 4)	1.787	(1.69,1.92)	0.244	(0.174,0.303)
HD9_CM	0.414	(0.414,0.414)	4.908	(4.862,4.960)	2.642	(2.612,2.678)	4	(4, 4)	2.451	(2.43,2.47)	0.943	(0.935,0.952)
HD9_TN	0.145	(0.043,0.145)	3.389	(3.024,3.408)	1.531	(1.287,1.542)	5	(5, 7)	1.363	(1.07,1.37)	0.281	(0.273,0.691)

Table L: Estimated parameter values and 95% confidence intervals, generated from 1,000 bootstrap replicates, for the type 1 diabetes patients described in Relationships among sorted $CD4^{+}$ T cell subtypes in individuals with type 1 diabetes and healthy donors.

	ϕ		α		β		u		σ		ξ	
ID	est.	95% CI	est.	95% CI	est.	95% CI	est.	95% CI	est.	95% CI	est.	95% CI
T1D1_CM	0.397	(0.397,0.397)	4.956	(4.894,5.017)	2.491	(2.452,2.532)	4	(4, 4)	1.889	(1.87,1.91)	0.676	(0.667,0.685)
T1D1_TN	0.245	(0.245,0.245)	3.760	(3.726,3.794)	1.940	(1.915,1.964)	4	(4, 4)	1.225	(1.21,1.23)	0.118	(0.111,0.125)
T1D1_Treg	0.049	(0.049,0.049)	1.004	(0.945,1.062)	1.304	(1.226,1.381)	4	(4, 4)	1.475	(1.35,1.61)	0.789	(0.716,0.864)
T1D1_Tscm	0.047	(0.047,0.047)	2.319	(2.235,2.422)	2.341	(2.249,2.448)	4	(4, 4)	1.276	(1.17,1.37)	0.175	(0.117,0.231)
T1D10_CM	0.359	(0.269,0.359)	2.704	(2.311,2.728)	1.125	(0.838,1.141)	5	(5, 6)	2.580	(2.56,2.68)	0.902	(0.895,1.071)
T1D10_TN	0.100	(0.037,0.100)	6.406	(5.056,6.456)	4.105	(3.035,4.141)	4	(4, 5)	0.892	(0.77,0.91)	0.077	(0.058,0.136)
T1D11_CM	0.349	(0.349,0.349)	5.141	(5.095,5.189)	2.840	(2.809,2.872)	4	(4, 4)	2.068	(2.05,2.09)	0.860	(0.853,0.869)
T1D11_TN	0.044	(0.002,0.002)	10.321	(2.458,2.521)	11.605	(2.772,2.823)	3	(4, 4)	0.105	(0.53,0.87)	0.604	(0.418,0.773)
T1D12_CM	0.214	(0.214,0.214)	5.639	(5.578,5.700)	3.614	(3.570,3.659)	4	(4, 4)	1.818	(1.78,1.86)	1.089	(1.072,1.106)
T1D12_TN	0.219	(0.219,0.219)	5.047	(5.018,5.076)	2.800	(2.780,2.821)	4	(4, 4)	1.142	(1.13,1.15)	0.230	(0.223,0.237)
T1D13_CM	0.229	(0.229,0.229)	4.984	(4.925,5.038)	3.205	(3.162,3.249)	4	(4, 4)	1.982	(1.95,2.02)	1.097	(1.081,1.114)
T1D13_TN	0.211	(0.211,0.211)	4.575	(4.547,4.601)	2.565	(2.545,2.585)	4	(4, 4)	1.189	(1.18,1.20)	0.114	(0.108,0.121)
T1D14_CM	0.248	(0.248,0.248)	5.393	(5.338,5.451)	3.328	(3.290,3.370)	4	(4, 4)	1.851	(1.82,1.88)	0.883	(0.870,0.897)
T1D14_TN	0.205	(0.205,0.205)	4.837	(4.809,4.863)	2.708	(2.688,2.727)	4	(4, 4)	1.141	(1.13,1.15)	0.105	(0.099,0.111)
T1D2_CM	0.084	(0.084,0.140)	1.725	(1.709,1.820)	0.861	(0.851,0.944)	6	(5, 6)	1.906	(1.60,1.94)	0.641	(0.543,0.667)
T1D2_TN	0.282	(0.282,0.282)	3.897	(3.865,3.932)	1.979	(1.957,2.004)	4	(4, 4)	1.429	(1.42,1.44)	0.110	(0.104,0.116)
T1D2_Treg	0.053	(0.031,0.053)	0.947	(0.883,0.983)	0.836	(0.760,0.875)	5	(5, 7)	1.596	(1.49,2.18)	0.673	(0.546,0.858)
T1D2_Tscm	0.005	(0.005,0.005)	2.526	(2.379,2.719)	3.450	(3.276,3.676)	4	(4, 4)	0.823	(0.61,1.08)	0.044	(-0.170,0.218)
T1D3_CM	0.180	(0.180,0.180)	2.716	(2.693,2.739)	0.833	(0.823,0.842)	7	(7, 7)	2.789	(2.74,2.83)	0.807	(0.793,0.821)
T1D3_TN	0.011	(0.011,0.111)	3.695	(3.679,4.760)	2.114	(2.104,2.966)	6	(4, 6)	0.321	(0.30,0.90)	0.481	(-0.016,0.517)
T1D3_Treg	0.071	(0.047,0.071)	1.103	(1.037,1.129)	0.916	(0.835,0.943)	5	(5, 6)	1.771	(1.69,2.07)	0.983	(0.941,1.148)
T1D3_Tscm	0.013	(0.013,0.013)	2.728	(2.616,2.843)	3.307	(3.178,3.441)	4	(4, 4)	1.007	(0.85,1.21)	0.348	(0.159,0.508)
T1D4_CM	0.220	(0.135,0.220)	3.809	(3.258,3.846)	1.696	(1.329,1.718)	5	(5, 6)	1.467	(1.29,1.48)	0.748	(0.737,1.088)
T1D4_TN	0.103	(0.103,0.103)	4.094	(4.062,4.127)	2.606	(2.580,2.630)	4	(4, 4)	0.780	(0.77,0.79)	0.107	(0.092,0.119)
T1D4_Treg	0.036	(0.023,0.063)	0.904	(0.829,1.007)	1.012	(0.910,1.158)	5	(4, 6)	1.704	(1.36,1.82)	0.741	(0.601,1.046)
T1D4_Tscm	0.032	(0.032,0.032)	2.216	(2.155,2.276)	2.476	(2.410,2.542)	4	(4, 4)	1.045	(0.98,1.12)	0.332	(0.274,0.387)
T1D5_CM	0.354	(0.354,0.354)	5.025	(4.962,5.093)	2.612	(2.570,2.659)	4	(4, 4)	1.660	(1.64,1.68)	0.673	(0.663,0.685)
T1D5_TN	0.040	(0.040,0.040)	2.873	(2.858,2.891)	1.060	(1.052,1.067)	7	(7, 7)	0.786	(0.76,0.81)	0.237	(0.216,0.259)
T1D5_Treg	0.026	(0.016,0.050)	0.942	(0.876,1.009)	1.100	(1.009,1.200)	5	(4, 6)	1.479	(1.23,1.58)	0.700	(0.579,1.001)
T1D5_Tscm	0.018	(0.018,0.018)	2.046	(1.938,2.165)	2.606	(2.473,2.740)	4	(4, 4)	0.935	(0.79,1.10)	0.451	(0.318,0.589)
T1D6_CM	0.375	(0.237,0.375)	4.967	(3.829,5.028)	2.521	(1.678,2.561)	4	(4, 5)	1.665	(1.65,1.69)	0.608	(0.600,0.788)
T1D6_TN	0.033	(0.033,0.088)	3.757	(3.744,4.173)	1.778	(1.770,2.062)	6	(5, 6)	0.456	(0.45,0.91)	0.338	(0.015,0.350)
T1D6_Treg	0.033	(0.033,0.058)	0.872	(0.850,0.953)	1.020	(0.992,1.141)	5	(4, 5)	1.918	(1.33,1.99)	0.880	(0.762,0.938)
T1D6_Tscm	0.025	(0.025,0.025)	2.099	(1.987,2.223)	2.434	(2.307,2.574)	4	(4, 4)	0.965	(0.83,1.11)	0.293	(0.189,0.393)
T1D7_CM	0.181	(0.108,0.311)	3.864	(3.364,4.932)	1.793	(1.453,2.599)	5	(4, 6)	1.365	(1.18,1.41)	0.712	(0.518,1.063)
T1D7_TN	0.232	(0.232,0.232)	4.428	(4.390,4.468)	2.374	(2.348,2.401)	4	(4, 4)	1.291	(1.28,1.30)	0.100	(0.093,0.107)
T1D7_Treg	0.063	(0.038,0.063)	1.113	(1.056,1.133)	0.685	(0.629,0.702)	6	(6, 7)	1.744	(1.65,3.71)	0.943	(0.714,0.987)
T1D7_Tscm	0.074	(0.074,0.074)	3.439	(3.367,3.516)	3.002	(2.933,3.071)	4	(4, 4)	1.360	(1.30,1.42)	0.125	(0.094,0.154)
T1D8_CM	0.059	(0.033,0.059)	1.211	(1.175,1.234)	0.894	(0.858,0.917)	5	(5, 6)	1.428	(1.37,1.92)	0.601	(0.568,0.695)
T1D8_TN	0.240	(0.240,0.240)	4.194	(4.151,4.236)	2.221	(2.192,2.249)	4	(4, 4)	1.290	(1.28,1.30)	0.038	(0.028,0.046)
T1D8_Treg	0.031	(0.020,0.054)	0.875	(0.771,1.000)	1.075	(0.929,1.259)	5	(4, 6)	1.662	(1.06,1.75)	0.540	(0.400,1.001)
T1D8_Tscm	0.070	(0.070,0.070)	2.869	(2.776,3.015)	2.608	(2.520,2.738)	4	(4, 4)	1.451	(1.33,1.54)	0.200	(0.152,0.246)
T1D9_CM	0.313	(0.313,0.313)	6.595	(6.539,6.652)	3.974	(3.936,4.012)	4	(4, 4)	2.316	(2.29,2.34)	1.172	(1.160,1.182)
T1D9_TN	0.019	(0.019,0.019)	2.466	(2.437,2.496)	2.335	(2.307,2.364)	4	(4, 4)	0.140	(0.12,0.16)	0.667	(0.600,0.739)

Table M: The thresholds and shape parameters inferred with the Desponds et al. model and the ecological estimators computed on the healthy donor versus type 1 diabetes data. Additionally, the proportion of highly stimulated clones, as computed from the spliced model.

ID	Desponds u_d	Desponds α_d	richness	Shannon	clonality	prop. highly stimulated
HD1_CM	15	1.266	250236	11.583	0.068	0.801
HD1_TN	14	4.018	440905	12.798	0.015	0.626
HD1_Treg	6	1.319	42987	10.232	0.041	0.291
HD1_Tscm	7	2.429	26163	9.842	0.032	0.468
HD10_CM	6	1.034	488176	12.025	0.082	0.811
HD10_TN	19	3.789	1276177	13.907	0.011	0.148
HD11_CM	131	1.657	250109	11.256	0.094	0.686
HD11_TN	32	1.805	878903	13.514	0.013	0.472
HD12_CM	8	1.069	421593	11.623	0.103	0.625
HD12_TN	5	1.961	337679	12.652	0.006	0.006
HD13_CM	195	1.719	386343	11.431	0.111	0.774
HD13_TN	15	1.882	683043	13.315	0.009	0.074
HD14_CM	12	1.062	444150	10.899	0.162	0.732
HD14_TN	8	2.395	637046	13.199	0.012	0.192
HD2_CM	9	1.167	266244	11.301	0.095	0.725
HD2_TN	8	3.334	610156	13.197	0.009	0.300
HD2_Treg	8	1.056	103075	10.981	0.049	0.265
HD2_Tscm	42	1.568	142717	11.558	0.026	0.473
HD3_CM	19	1.379	230115	11.469	0.071	0.856
HD3_TN	25	3.291	555941	13.089	0.011	0.532
HD3_Treg	7	1.266	64726	10.647	0.039	0.300
HD3_Tscm	7	2.945	69621	10.917	0.021	0.322
HD4_CM	17	1.483	257561	11.807	0.052	0.654
HD4_TN	17	4.080	620855	13.168	0.013	0.587
HD4_Treg	12	1.449	253386	12.239	0.016	0.047
HD4_Tscm	11	3.388	32337	10.133	0.024	0.348
HD5_CM	10	1.890	67717	10.779	0.031	0.274
HD5_TN	12	3.499	389324	12.663	0.016	0.681
HD5_Treg	8	1.297	15936	9.567	0.011	0.024
HD5_Tscm	4	2.247	26262	9.965	0.021	0.266
HD6_CM	9	1.121	319165	11.926	0.059	0.504
HD6_TN	11	2.438	649422	13.280	0.008	0.160
HD6_Treg	10	1.256	74227	10.716	0.044	0.244
HD6_Tscm	14	4.615	191030	11.939	0.018	0.365
HD7_CM	13	1.328	292927	11.714	0.069	0.724
HD7_TN	15	3.843	519906	12.994	0.013	0.523
HD7_Treg	6	1.376	48104	10.463	0.029	0.225
HD7_Tscm	21	1.864	152471	11.570	0.031	0.477
HD8_CM	17	1.570	233334	11.725	0.051	0.658
HD8_TN	46	0.711	252570	11.826	0.049	0.118
HD8_Treg	6	1.502	10105	9.065	0.017	0.068
HD8_Tscm	4	1.982	22158	9.647	0.036	0.396
HD9_CM	12	1.063	409677	11.561	0.105	0.838
HD9_TN	8	2.266	682897	13.273	0.012	0.309

ID	Desponds u_d	Desponds α_d	richness	Shannon	clonality	prop. highly stimulated
T1D1_CM	11	1.292	297671	11.919	0.054	0.730
T1D1_TN	13	4.007	486446	12.961	0.010	0.431
T1D1_Treg	6	1.247	39331	10.157	0.040	0.248
T1D1_Tscm	5	2.633	37705	10.386	0.014	0.148
T1D10_CM	11	1.083	515054	11.932	0.093	0.792
T1D10_TN	9	2.041	331364	12.620	0.007	0.202
T1D11_CM	138	1.846	493471	12.137	0.074	0.747
T1D11_TN	5	1.859	235542	12.289	0.007	0.099
T1D12_CM	7	0.944	211711	11.018	0.102	0.671
T1D12_TN	17	1.997	906973	13.567	0.011	0.402
T1D13_CM	9	0.953	211660	10.828	0.117	0.714
T1D13_TN	29	2.240	906527	13.589	0.009	0.385
T1D14_CM	13	1.254	253210	11.547	0.072	0.646
T1D14_TN	30	2.881	986439	13.681	0.009	0.372
T1D2_CM	22	1.732	178423	11.727	0.030	0.294
T1D2_TN	17	3.904	617242	13.187	0.011	0.486
T1D2_Treg	8	1.535	46142	10.393	0.032	0.235
T1D2_Tscm	5	3.776	26235	10.104	0.007	0.019
T1D3_CM	11	1.380	303193	12.008	0.049	0.514
T1D3_TN	10	2.645	457704	12.938	0.007	0.031
T1D3_Treg	9	1.098	95954	10.729	0.065	0.380
T1D3_Tscm	5	2.249	41933	10.525	0.011	0.053
T1D4_CM	9	1.272	269762	11.991	0.041	0.519
T1D4_TN	12	2.790	462254	12.940	0.008	0.209
T1D4_Treg	7	1.364	46762	10.395	0.033	0.198
T1D4_Tscm	10	2.008	88503	11.248	0.013	0.111
T1D5_CM	8	1.230	231629	11.729	0.051	0.680
T1D5_TN	13	3.726	444034	12.884	0.009	0.096
T1D5_Treg	9	1.459	71850	10.911	0.024	0.143
T1D5_Tscm	8	2.033	27609	10.109	0.011	0.069
T1D6_CM	10	1.254	277784	11.939	0.048	0.688
T1D6_TN	15	2.754	648213	13.282	0.007	0.079
T1D6_Treg	8	1.194	76123	10.790	0.040	0.219
T1D6_Tscm	6	2.615	23532	9.946	0.012	0.085
T1D7_CM	9	1.309	263093	12.060	0.034	0.446
T1D7_TN	18	3.413	482536	12.956	0.010	0.415
T1D7_Treg	10	1.357	89188	10.890	0.045	0.302
T1D7_Tscm	9	4.021	80487	11.140	0.014	0.202
T1D8_CM	14	1.866	114290	11.353	0.025	0.222
T1D8_TN	10	4.151	407562	12.793	0.010	0.421
T1D8_Treg	9	1.584	25755	9.899	0.025	0.157
T1D8_Tscm	8	3.199	42034	10.472	0.016	0.205
T1D9_CM	16	1.013	377076	11.202	0.128	0.816
T1D9_TN	8	2.393	345571	12.655	0.008	0.052

References

1. J Antoch MH, Jarušková D. Change point detection. 5th ERS IASC Summer School. 2000;.
2. Hsu MS, Sedighim S, Wang T, Antonios JP, Everson RG, Tucker AM, et al. TCR sequencing can identify and track glioma-infiltrating T cells after DC vaccination. Cancer Immunol Res. 2016;4(5):412–418.
