# Supplementary material for: powerTCR: A model-based approach to comparative analysis of the clone size distribution of the T cell receptor repertoire
Source: PLoS Comput Biol. 2018 Nov 28;14(11):e1006571. doi: 10.1371/journal.pcbi.1006571 (PMC6287877; doi:10.1371/journal.pcbi.1006571)
Supplement: S5 Text — By comparing results from our full model to those from only our tail model, we observe empirically the gains from including the full clone size distribution. (PDF) [file pcbi.1006571.s005.pdf]

Supplementary file 5 — Comparative analysis using the GPD

We considered the implications of using only data above the threshold from our spliced model for comparative analysis. Doing so can provide some insight into the relative contribution of the bulk part of the model to clustering accuracy. We first extracted the tail portion of each fit estimated using the spliced model. We then computed pairwise JSD between each tail fit analagous to all other comparative analyses. Clustering results, executed exactly as in the main text, are presented in Fig A. Empirically, we observe that incorporating the entire distribution, as opposed to only the tail part, contributes to better clustering outcome.

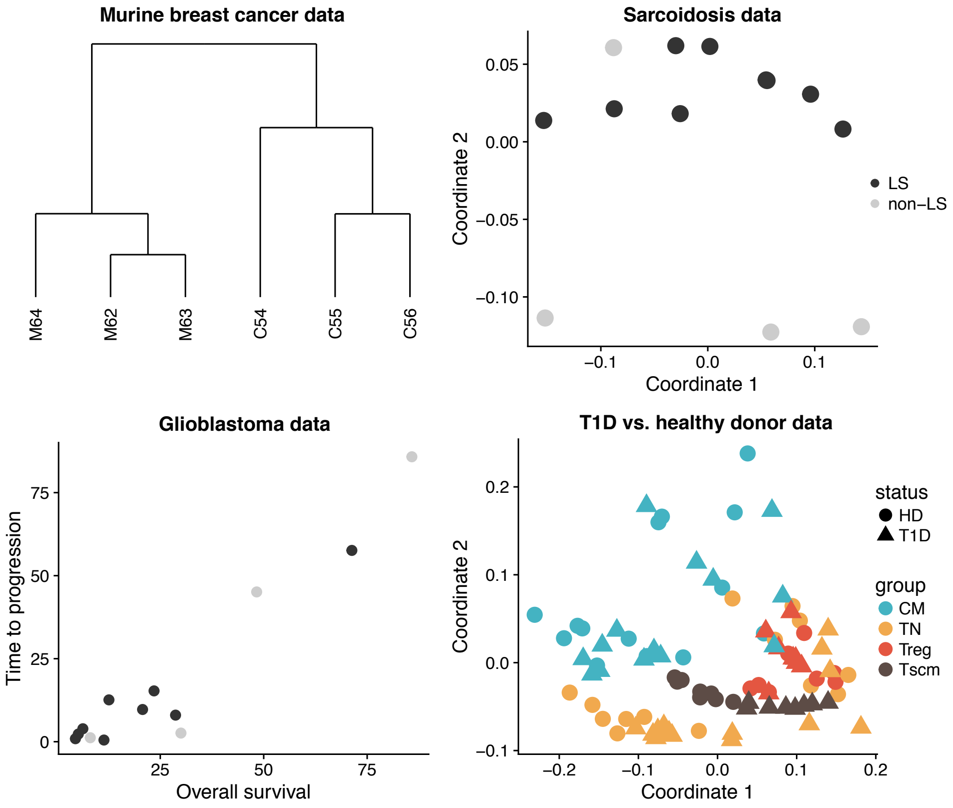

Figure A: Clustering results matching analyses in the main text, using only the data at and above the estimated threshold using our spliced model.
