# Supplementary material for: powerTCR: A model-based approach to comparative analysis of the clone size distribution of the T cell receptor repertoire
Source: PLoS Comput Biol. 2018 Nov 28;14(11):e1006571. doi: 10.1371/journal.pcbi.1006571 (PMC6287877; doi:10.1371/journal.pcbi.1006571)

Supplementary file 6 — Dendrograms for downsampling study

We downsampled mouse tumor data to 80, 60, 40, and 20% of total reads. We used JSD to compute pairwise distances between the samples for our model and the Desponds et al. model at each downsample level and did hierarchical clustering using Ward's method. The dendrograms for each model at each downsample level are presented here.

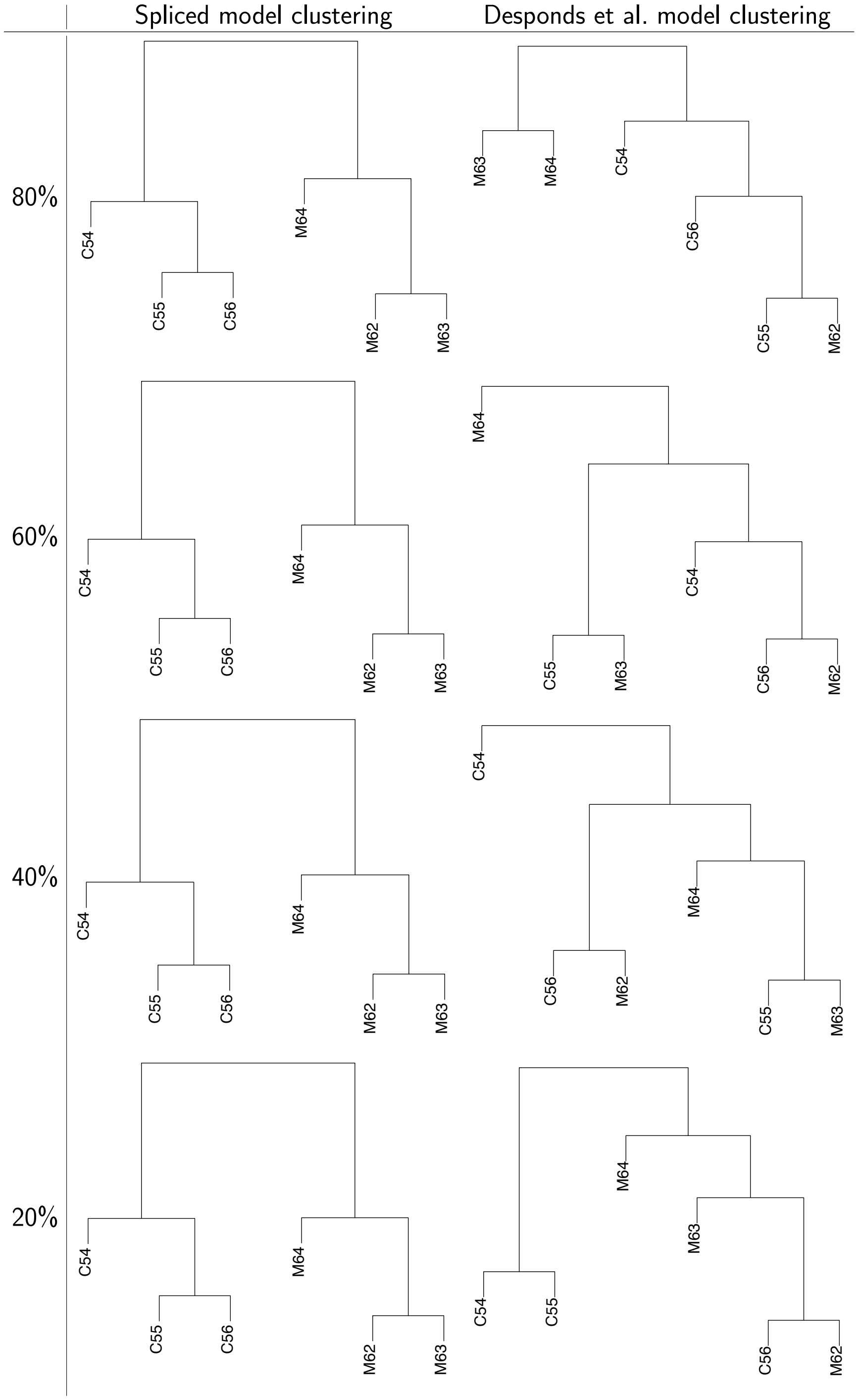

Supplement: S6 Text — We downsampled mouse tumor data to 80, 60, 40, and 20% of total reads. We used JSD to compute pairwise distances between the samples for our model fits and the Desponds et al. model fits at each downsample level and did hierarchical clustering using Ward’s method. The dendrograms for each model at each downsample level are presented here. (PDF) [file pcbi.1006571.s006.pdf]
