# Supplementary material for: powerTCR: A model-based approach to comparative analysis of the clone size distribution of the T cell receptor repertoire
Source: PLoS Comput Biol. 2018 Nov 28;14(11):e1006571. doi: 10.1371/journal.pcbi.1006571 (PMC6287877; doi:10.1371/journal.pcbi.1006571)
Supplement: S7 Text — Clustering dendrograms generated on pre- and post-treatment glioblastoma samples. Groupings presented for the post-treatment samples here correspond to the colored groupings in Fig 5. (PDF) [file pcbi.1006571.s007.pdf]

Supplementary file 7 — Clustering of pre- and post-treatment glioblastoma patient samples

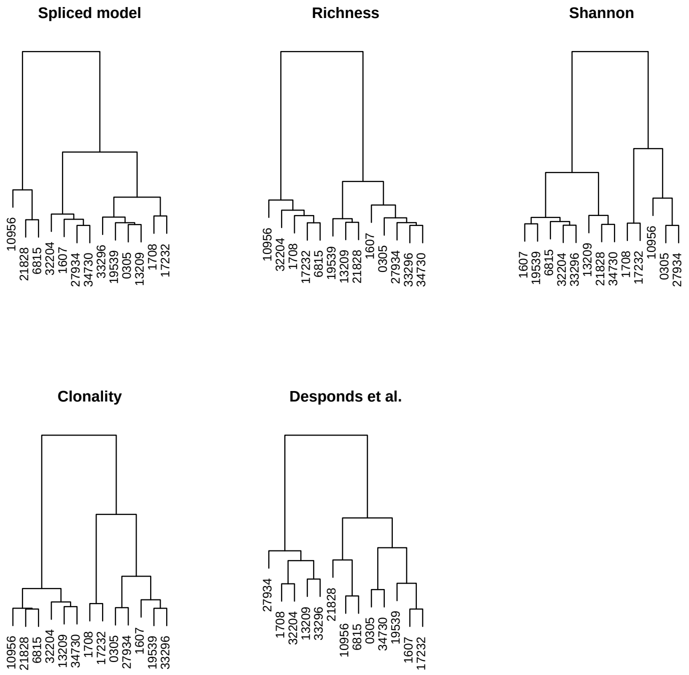

Figure A: Dendrograms displaying the full hierarchical clustering of pre-treatment PBMC samples from glioblastoma patients. No trend was evident among pre-treatment patients using any of the considered methods.

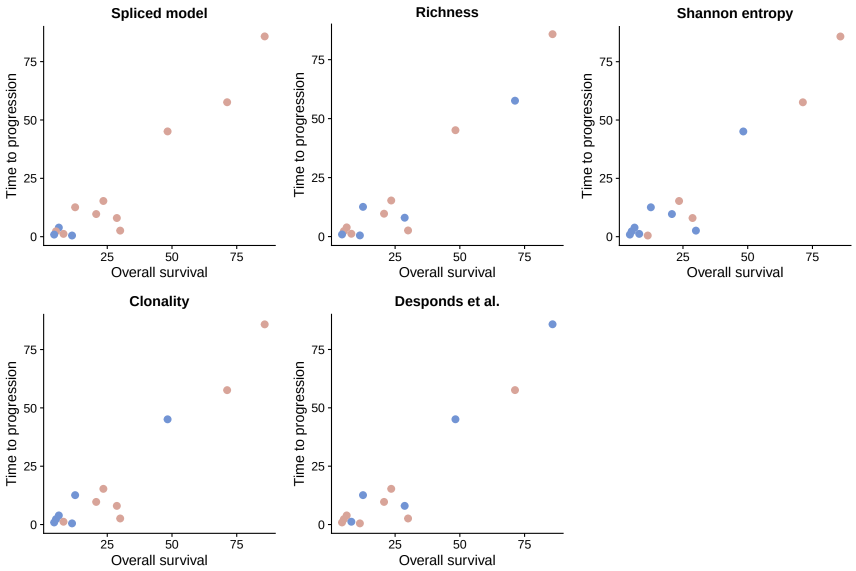

Figure B: The hierarchical clustering of pre-treatment PBMC samples can be split into two groups. Plotting patients by OS versus TTP and coloring by groups does not reveal a trend using any of the proposed methods.

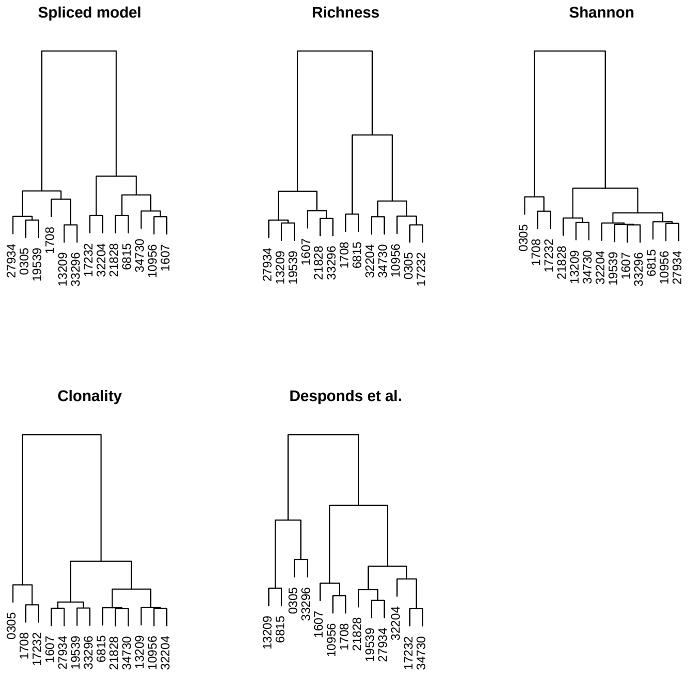

Figure C: Dendrograms displaying the full hierarchical clustering of post-treatment PBMC samples from glioblastoma patients. The clustering here corresponds to the groupings presented in the main text.
