# Supplementary material for: powerTCR: A model-based approach to comparative analysis of the clone size distribution of the T cell receptor repertoire
Source: PLoS Comput Biol. 2018 Nov 28;14(11):e1006571. doi: 10.1371/journal.pcbi.1006571 (PMC6287877; doi:10.1371/journal.pcbi.1006571)
Supplement: S8 Text — This plot calls out patients 33296 and 17232 from the glioblastoma patients. Patient 33296 incorrectly clustered with the individuals with favorable clinical outcome, while patient 17232 incorrectly clustered with individuals with unfavorable clinical outcome. (PDF) [file pcbi.1006571.s008.pdf]

Supplementary file 8 — Clone size distributions of glioblastoma patients

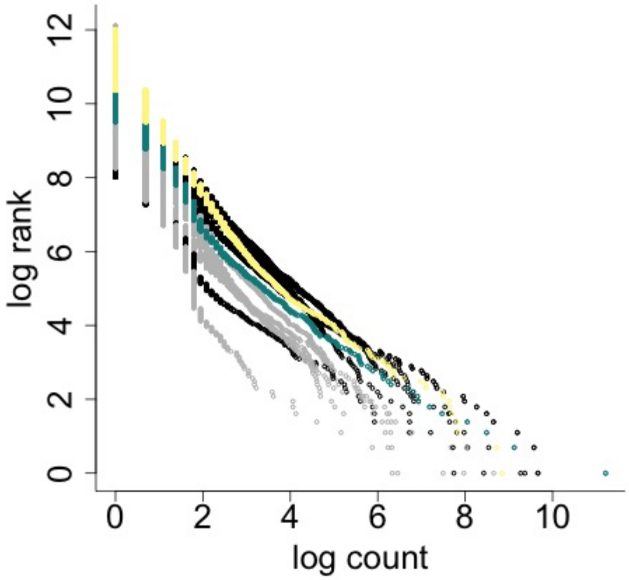

Figure A: Two anomalous patients appeared in the glioblastoma study. Here, we plot the clone size distributions of every post-treatment PBMC sample. Patients colored in black had overall better clinical outcome, while patients in grey had worse clinical outcome. The yellow patient, patient 33296, had among the poorest clinical outcome but clustered (using the spliced threshold model) in a group with the patients with better clinical outcome. The blue patient, patient 17232, had among the best clinical outcome but clustered in a group with the patients with poorer clinical outcome.
